# Effect of lymph node dissection on stage-specific survival in patients with upper urinary tract urothelial carcinoma treated with nephroureterectomy

**DOI:** 10.1186/s12885-019-6364-z

**Published:** 2019-12-12

**Authors:** Ting-Shuai Zhai, Liang Jin, Zhen Zhou, Xiang Liu, Huan Liu, Wei Chen, Jing-Yi Lu, Xu-Dong Yao, Li-Ming Feng, Lin Ye

**Affiliations:** 1 Department of Urology, Shanghai Tenth People’s Hospital of Tongji University, School of Medicine, Shanghai, 200072 China; 2 Department of Urology, Shawan People’s Hospital, Shawan, Xinjiang 832100 China; 3 Nanjing Medical University, Nanjing, 210000 China; 4 Department of Urology, Shanghai Putuo District People’s Hospital of Tongji University, School of Medicine, Shanghai, 200333 China; 5 Department of Urology, Karamay Central Hospital, Karamay, Xinjiang, 834000 China

**Keywords:** Lymph node dissection, Neoplasm staging, SEER program, Survival analysis, Upper urinary tract urothelial carcinoma

## Abstract

**Background:**

We aimed to estimate the stage-specific impact of lymph node dissection (LND) on survival for upper urinary tract urothelial carcinoma (UTUC) patients treated with nephroureterectomy (NU).

**Methods:**

Overall, 7278 UTUC patients undergoing NU within the SEER database from 2004 to 2015 were identified. Kaplan-Meier plots illustrated overall survival (OS) and cancer-specific survival (CSS) rates according to LND status. Multivariable Cox regression analyses assessed the effect of LND on OS and CSS rates stratified by pathological tumor stage.

**Results:**

LND was performed in 26.9% of patients, and in 18.6, 23.3, 31.2 and 45.9% for pT1, pT2, pT3 and pT4 patients, respectively (*P* <  0.001). In multivariable Cox regression analyses, LND was associated with a higher OS or CSS in UTUC patients with pT3 and pT4 disease (all *P* <  0.05), but failed to achieve independent predictor status in patients with pT1 and pT2 disease (all *P* > 0.05). LND with 1 to 3 regional lymph nodes removed was prone to a higher OS or CSS only in pT4 compared to no LND (both *P* <  0.01). LND with 4 or more regional lymph nodes removed predisposed to a higher OS or CSS in pT3 or pT4 (all *P* <  0.05).

**Conclusions:**

The beneficial effect of LND especially LND with 4 or more regional lymph nodes removed on survival was evident in pT3/4 patients. LND can be considered for pT3 and pT4, for pT1/2 remains to be seen, both of which will be verified by further prospective studies.

## Background

Upper urinary tract urothelial carcinomas (UTUCs) are rare tumors, which account for only 5–10% of urothelial carcinomas (UCs) [1, 2]. Nephroureterectomy (NU) is the standard surgical management for high-risk UTUC [2]. At NU, lymph node dissection (LND) allows for optimal tumor staging and improves the detection accuracy of lymph node metastases (LNM) [3, 4]. Moreover, LND might have a potential benefit on survival outcomes for advanced-stage UTUC patients [5]. However, there are some disadvantages resulting from LND such as long surgical time and increased postoperative complications [6].

Based on the observations above, the European Association of Urology practice guidelines recommended a lymphadenectomy in patients with high-risk tumors while the strength rating was weak [2]. LND was omitted in a large amount of UTUC patients. A recent research observed that LND was performed in only 36% of UTUC patients who underwent NU and LND was performed more frequently in patients treated with open NU [7]. LND omission in these patients might decrease survival rate according to the literatures. To date, the curative role of LND remains debated. Few previous studies have evaluated the effect of LND on survival for UTUC patients with different tumor stage separately. To resolve this issue, we tested the impact of LND at NU on overall survival (OS) and cancer-specific survival (CSS). Our hypothesis stated that LND may benefit OS and CSS, which is consistent across all tumor stages.

## Methods

### Study population

Within the Surveillance, Epidemiology and End Results (SEER) database from 2004 to 2015, we identified 7278 patients with histologically confirmed UTUC who underwent NU with (1 to 3 / 4 or more regional lymph nodes removed) or without LND. Only patients with non-metastatic transitional cell carcinoma were considered. None of the patients in this study had prior history of bladder cancer. No patient with node positive disease preoperatively was included. Patients with unknown tumor stage, tumor grade and unknown LND status were excluded.

### Definition of variables for analyses

Patients were stratified according to presence or absence of LND. Covariates consisted of age at diagnosis, gender (male, female), race (white, other), marital status (married, unmarried, unknown), primary site (renal pelvis, ureter), laterality (left, right, paired), tumor size (≤ 2 cm and > 2 cm), tumor stage (T1, T2, T3, T4), tumor grade (I, II, III, IV) and year of surgery (2004–2007, 2008–2011, 2012–2015).

### Statistical analysis

Continuous data are reported as mean ± s.d. and were analyzed by Student’s t test. Categorical variables were compared using χ^2^ test or Fisher’s exact test, as appropriate. Kaplan-Meier plots graphically explored overall survival (OS) and cancer-specific survival (CSS) curves. Our Cox regression analyses included three steps. Firstly, cox regression analyses tested the impact of LND (LND vs no LND) on OS and CSS. Secondly, we used cox regression analyses to estimate the relationship between LND extent and survival. Limited LND was defined as removing 1 to 3 regional lymph nodes, and extended LND was defined as removing ≥4 regional lymph nodes. Thirdly, cox regression analyses were used to test the effect of lymph node stage (pN0 vs pNx vs pN1–3) on OS and CSS. The study population was divided into lymph node-negative (N0), lymph node-positive (N1–3) (LNM), and regional lymph nodes not removed (Nx) groups. In all multivariable analyses, covariates comprised age, gender (male vs female), race (white vs other), tumor location (renal pelvis vs ureter), laterality (left vs right), tumor size (≤ 2 cm vs > 2 cm), pathological tumor stage (pT1 vs pT2 vs pT3 vs pT4), lymph node stage (N0 vs N1 vs N2 vs N3) (AJCC 6th ed), tumor grade (grade I vs grade II vs grade III vs grade IV), chemotherapy (No/Unknown vs Yes) and year of surgery categories (2004–2007 vs 2008–2011 vs 2012–2015). Finally, all the aforementioned analyses were repeated for each tumor stage. The 95% CIs were calculated and *P* <  0.05 was considered statistically significant. SPSS (IBM SPSS Statistics 25) was used for analyses.

## Results

### General characteristics

From 2004 to 2015, 7278 patients (median age 73 years, range: 22–101) underwent NU for UTUC within the SEER database (Table 1). The majority were male (59.0%), white race (88.2), married status (60.7%), renal pelvis (69.1%), left Laterality (50.4%) and big tumor size (73.6%). Overall, 2279 patients harbored T1 (31.3%) vs 1353 T2 (18.6%) vs 3075 T3 (42.3%) vs 571 T4 (7.8%) stage and 292 patients harbored grade I (4.0%) vs 1102 grade II (15.1%) vs 2096 grade III (28.8%) vs 3788 grade IV (52.0%). Among them, 1258 (17.3%) patients received adjuvant chemotherapy. 5317 (73.1%) patients underwent NU without LND, 1961 (26.9%) patients received LND.

**Table 1:** Characteristics for UTUC patients stratified by lymph node dissection

Characteristic	Total^a^	No LND^a^	LND^a^	*P value*^b^
	No. (%)	No. (%)	No. (%)	
Total	7278 (100)	5317 (73.1)	1961 (26.9)	
Age (years) ^c^				< 0.001
Mean ± SD	71.9 ± 10.8	72.4 ± 10.8	70.7 ± 10.6	
Median	73	74	72	
Range	22–101	22–101	34–96	
Gender				0.904
Male	4295 (59.0)	3140 (43.1)	1155 (15.9)	
Female	2983 (41.0)	2177 (29.9)	806 (11.1)	
Race				< 0.001
White	6419 (88.2)	4744 (65.2)	1675 (23.0)	
Other	859 (11.8)	573 (7.9)	286 (3.9)	
Marital status				0.284
Married	4421 (60.7)	3205 (44.0)	1216 (16.7)	
Unmarried	2588 (35.6)	1919 (26.4)	669 (9.2)	
Unknown	269 (3.7)	193 (2.7)	76 (1.0)	
Primary site				0.574
Renal pelvis	5032 (69.1)	3686 (50.6)	1346 (18.5)	
Ureter	2246 (30.9)	1631 (22.4)	615 (8.5)	
Laterality				< 0.001
Left	3665 (50.4)	2576 (35.4)	1089 (15.0)	
Right	3607 (49.6)	2736 (37.6)	871 (12.0)	
Paired	6 (< 1%)	5 (< 1%)	1 (< 1%)	
Tumor size				< 0.001
≤ 2 cm	1178 (16.2)	899 (12.4)	279 (3.8)	
> 2 cm	5357 (73.6)	3833 (52.7)	1524 (20.9)	
Unknown	743 (10.2)	585 (8.0)	158 (2.2)	
Grade				< 0.001
I	292 (4.0)	249 (3.4)	43 (0.6)	
II	1102 (15.1)	922 (12.7)	180 (2.5)	
III	2096 (28.8)	1492 (20.5)	604 (8.3)	
IV	3788 (52.0)	2654 (36.5)	1134 (15.6)	
T stage				< 0.001
T1	2279 (31.3)	1854 (25.5)	425 (5.8)	
T2	1353 (18.6)	1038 (14.3)	315 (4.3)	
T3	3075 (42.3)	2116 (29.1)	959 (13.2)	
T4	571 (7.8)	309 (4.2)	262 (3.6)	
Lymph node status status				–
pNx	5317 (73.1)	5317 (73.1)	–	
pN0	1296 (17.8)	–	1296 (17.8)	
pN1–3	665 (9.1)	–	665 (9.1)	
Chemotherapy				< 0.001
No/Unknown	6020 (82.7)	4635 (63.7)	1385 (19.0)	
Yes	1258 (17.3)	682 (9.4)	576 (7.9)	
Year of surgery				
2004–2007	2424 (33.3)	1865 (25.6)	559 (7.7)	< 0.001
2008–2011	2451 (33.7)	1803 (24.8)	648 (8.9)	
2012–2015	2403 (33.0)	1649 (22.7)	754 (10.4)	

^a^With percentages in parentheses; ^b^Fisher’s exact test or χ^2^ test, except ^c^Student’s t test. LND, Lymph Node Dissection

### Trends in LND and LNM

LND was more frequently performed in younger patients, non-white race, left laterality, bigger tumor (> 2 cm), higher grade and advanced tumor stage (all *P* <  0.001) (Table 1). Moreover, LND rate was increasing evidently from 2004 (23.5%) to 2015 (34.1%) (*P* <  0.001) (Additional file 1: Table S1 and Figure S1, supporting information). Of the 1961 patients receiving LND, 1108 (56.5%) were limited LND and 853 (43.5%) were extended LND (Additional file 1: Table S2).

In patients who received LND (*n* = 1961), the overall rate of lymph node metastasis (LNM, pN1–3) was 33.9%. For the same stage, LNM rates were 12.2, 20.3, 39.4 and 65.3% for pT1, pT2, pT3 and pT4 patients, respectively (*P* <  0.001). Of the 1961 patients, 56.5% underwent limited LND and 43.5% underwent extended LND, and the LNM rates were 31.4% vs 37.2%, respectively (*P* = 0.008). The rate of LNM in patients who received limited LND vs extended LND was respectively 9.4 vs 15.7% for pT1 (*P* = 0.048), 20.5 vs 20.1% for pT2 (*P* = 0.946), 36.3 vs 43.6% for pT3 (*P* = 0.022) and 35.1 vs 71.8% for pT4 (*P* = 0.058) (Additional file 1: Table S2). Extended LND was performed in respectively 44.9, 44.1, 43.1 and 42.0% (*P* = 0.867) for patients with pT1, pT2, pT3 and pT4 disease, respectively. While, extended LND was performed in 41.4, 35.8, 61.7 and 70.6% for patients with AJCC N0, N1, N2 and N3, respectively (*P* <  0.001) (Additional file 1: Table S3). The LND rates were 19.8, 86.5, 93.4 and 85.0% for AJCC N0, N1, N2 and N3 patients (*p* <  0.001) (Additional file 1: Table S4).

### Survival analyses according to LND status

The 5-year and 10-year OS and CSS rates for all pT stages patients according to LND status were shown in Table 2. For LND vs no LND patients, the 5-year OS rates and CSS rates were 41.5 vs 47.1% and 65.8 vs 74.3%. Stage-specific 5-year OS rates for LND vs no LND patients were 66.3 vs 64.5% for pT1, 50.1 vs 47.5% for pT2, 35.3 vs 36.2% for pT3, and 13.9 vs 12.8% for pT4 disease. The 5-year CSS rates for the same tumor stages were 87.6 vs 88.9%, 75.6 vs 78.6%, 59.2 vs 62.4% and 31.8 vs 31.2%, respectively.

**Table 2:** Life of the 5-year and 10-year overall survival and cancer-specific survival rates

	Overall survival rate (%)		Cancer-specific survival rate (%)	
	5 years	10 years	5 years	10 years
All stages				
LND status				
LND	41.5	26.2	65.8	60.3
No LND	47.1	29.2	74.3	67.8
LND extent				
No LND	47.1	29.2	74.3	67.8
Limited LND	39.2	24.3	63.5	57.1
Extended LND	44.9	28.5	69.0	65.0
Lymph node stage				
pN0	52.0	32.5	76.8	71.9
pNx	47.1	29.2	74.3	67.8
pN1–3	20.7	13.8	40.1	34.2
pT1 stage				
LND status				
LND	66.3	46.3	87.6	86.1
No LND	64.5	41.5	88.9	83.1
LND extent				
No LND	64.5	41.5	88.9	83.1
Limited LND	64.1	43.6	84.7	83.4
Extended LND	68.9	50.9	91.0	89.1
Lymph node stage				
pN0	69.3	47.8	90.3	89.5
pNx	64.5	41.5	88.9	83.1
pN1–3	44.1	34.7	64.7	58.2
pT2 stage				
LND status				
LND	50.1	27.3	75.6	67.3
No LND	47.5	27.6	78.6	69.9
LND extent				
No LND	47.5	27.6	78.6	69.9
Limited LND	48.6	29.7	73.3	64.0
Extended LND	52.0	23.8	79.1	71.9
Lymph node stage				
pN0	56.5	29.8	80.6	72.3
pNx	47.5	27.6	78.6	69.9
pN1–3	22.9	16.3	51.6	42.9
pT3 stage				
LND status				
LND	35.3	22.7	59.2	52.6
No LND	36.2	22.1	62.4	55.6
LND extent				
No LND	36.2	22.1	62.4	55.6
Limited LND	32.3	19.6	55.8	47.8
Extended LND	39.6	24.5	63.9	60.4
Lymph node stage				
pN0	43.8	26.6	69.3	63.3
pNx	36.2	22.1	62.4	55.6
pN1–3	22.2	14.6	42.1	35.2
PT4 stage				
LND status				
LND	13.9	8.6	31.8	30.2
No LND	12.8	4.8	31.2	28.8
LND extent				
No LND	12.8	4.8	31.2	28.8
Limited LND	15.1	8.3	36.6	NA
Extended LND	11.5	9.2	23.8	NA
Lymph node stage				
pN0	21.6	14.8	48.1	NA
pNx	12.8	4.8	31.2	28.8
pN1–3	9.9	4.5	19.6	NA

**Fig. 1:**
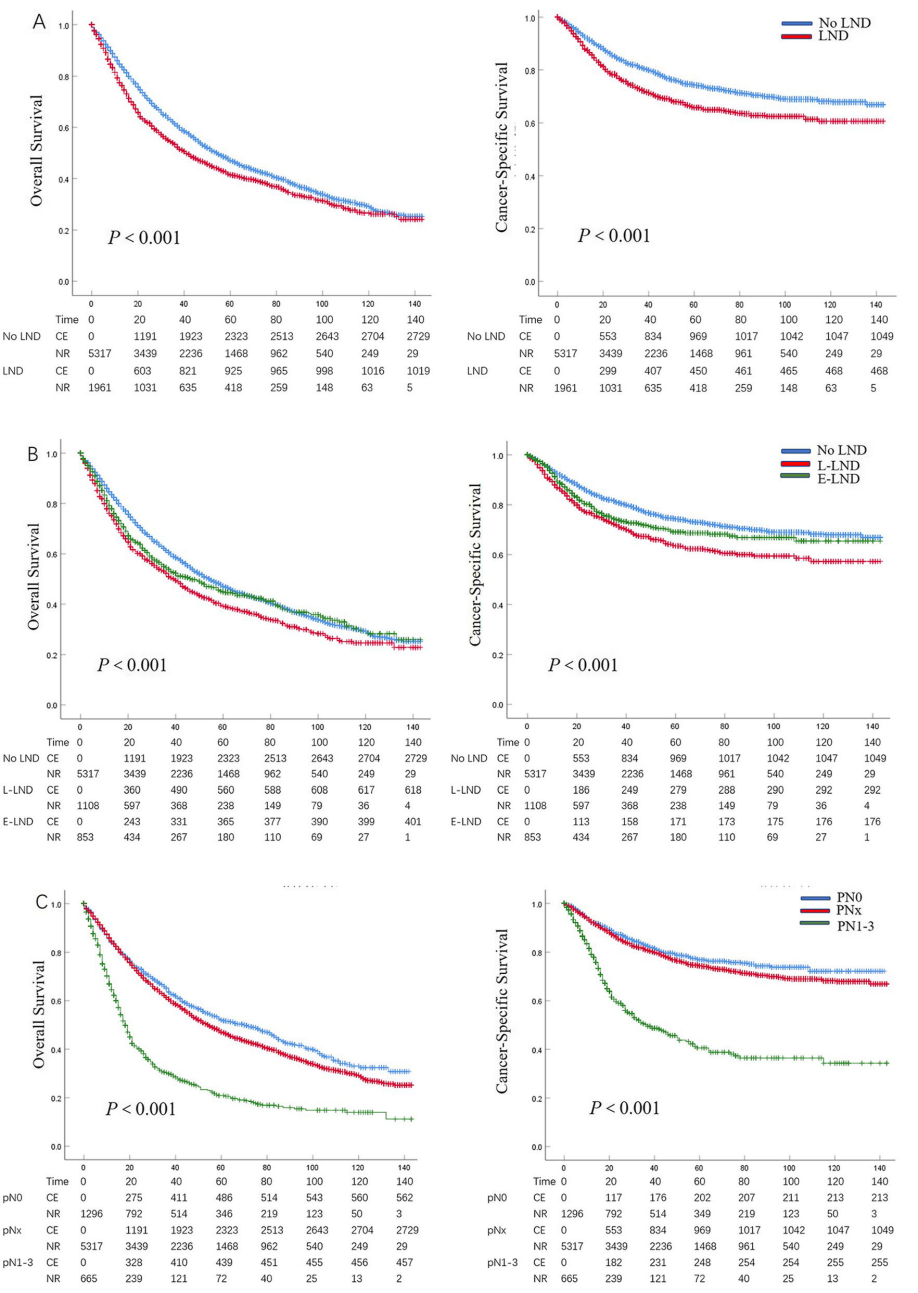
Kaplan-Meier plots depicting overall survival (OS) and cancer-specific survival (CSS), after stratification according to lymph node dissection (LND) status (**a**), LND extent (**b**), and lymph node stage (**c**) in 7278 patients treated with radical cystectomy between 2004 and 2015, within the Surveillance, Epidemiology and End Results database. C. E, cumulative number of events; N. R, number at risk; L-LND, Limited LND; E-LND, Extended LND

**Fig. 2:**
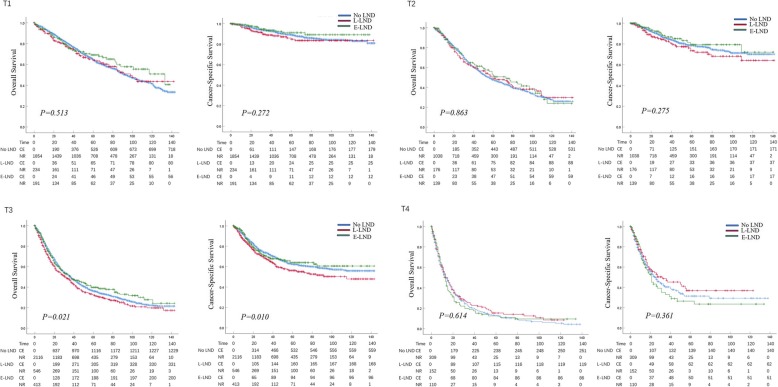
Kaplan-Meier plots depicting overall survival (OS) and cancer-specific survival (CSS) for pT1, pT2, pT3 and pT4 diseases, after stratification according to LND extent in 7278 patients treated with radical cystectomy between 2004 and 2015, within the Surveillance, Epidemiology and End Results database. C. E, cumulative number of events; N. R, number at risk; L-LND, Limited LND; E-LND, Extended LND

In patients with limited LND vs extended LND, the 5-year OS and CSS rates were 39.2 vs 44.9% and 63.5 vs 69.0%. In patients with pT1, pT2, pT3 and pT4 disease, the 5-year OS rates were 64.1 vs 68.9%, 48.6 vs 52.0%, 32.3 vs 39.6% and 15.1 vs 11.5% in patients with respectively limited LND vs extended LND. For the stage, the 5-year CSS rates were 84.7 vs 91.0%, 73.3 vs 79.1%, 55.8 vs 63.9% and 36.6 vs 23.8% in patients with respectively limited LND vs extended LND. In patients with pN0 vs pNx vs pN1–3 UTUC cancer, the 5-year OS and CSS rates were 52.0 vs 47.1 vs 20.7% and 76.8 vs 74.3 vs 40.1%. At 5 years, the adverse impact of LND omission (pNx) and of LNM (pN1–3) compared to pN0 was consistent across all tumor stages for both OS and CSS. Kaplan-Meier plots depicting OS and CSS rates, after stratification according to LND status, LND extent and lymph node stage were shown in Fig. 1a, Fig. 1b and Fig. 1c respectively. And Kaplan-Meier plots depicting OS and CSS for stage-specific disease stratifying T stage was shown in Fig. 2. The trend of 5-year OS and CSS rates across all tumor stage according to LND status was shown in Additional file 1: Figure S2 and Figure S3.

In multivariable COX regression analyses, patients who underwent LND had lower hazard ratio (HR) for OS (HR 0.87, *P* <  0.01) and CSS (HR 0.81, *P* <  0.01) rates compared to patients operated without LND (Tables 3 and 4). When stratifying according to tumor stage, the beneficial impact of LND on OS remained consistent for patients with pT3 or pT4 disease while disappeared for patients with pT1 or pT2 disease. Patients receiving extended LND (HR 0.83, *P* <  0.01) or limited LND (HR 0.90, *P* <  0.05) had evidently higher OS rate compared to no LND (Reference category). After the analyses were repeated across all pathological stages, the beneficial impact of extended LND on OS was only found in pT3 or pT4 stages (HR 0.80, *P* <  0.05, HR 0.77, *P* <  0.05 respectively). While, the protective effect of limited LND was only observed in patients with pT4 stage and its beneficial role seemed to be more evident than extended LND (limited LND vs extended LND: HR 0.73, *P* < 0.01 vs HR 0.77, *P* < 0.05). Similar results were found when CSS rates were tested (Tables 3 and 4). Finally, the OS rate was 1.2-fold and 2.2-fold higher (both *P* < 0.001) in pN0 patients relative to those with pNx and pN1–3 disease respectively (Tables 3 and 4), which were consistent when same analyses were performed across all tumor stages. Similar results were produced when CSS rates were tested.

**Table 3:** Multivariable Cox regression analyses predicting overall survival

	All stages	T1	T2	T3	T4
	Multivariable ^a^ HR (95% CI)	Multivariable ^b^ HR (95% CI)	Multivariable ^b^ HR (95% CI)	Multivariable ^b^ HR (95% CI)	Multivariable ^b^ HR (95% CI)
LND status					
No LND	1.00 (Ref.)	1.00 (Ref.)	1.00 (Ref.)	1.00 (Ref.)	1.00 (Ref.)
LND	0.87 (0.80–0.95)**	0.98 (0.80–1.19)	0.85 (0.70–1.04)	0.88 (0.78–0.99)*	0.74 (0.59–0.94)*
LND extent					
No LND	1.00 (Ref.)	1.00 (Ref.)	1.00 (Ref.)	1.00 (Ref.)	1.00 (Ref.)
Limited LND	0.90 (0.81–0.99)*	0.98 (0.78–1.25)	0.87 (0.69–1.11)	0.93 (0.81–1.07)	0.73 (0.57–0.94)**
Extended LND	0.83 (0.74–0.94)**	0.97 (0.73–1.30)	0.82 (0.61–1.09)	0.80 (0.68–0.95)*	0.77 (0.57–0.99)*
Lymph node stage					
pN0	1.00 (Ref.)	1.00 (Ref.)	1.00 (Ref.)	1.00 (Ref.)	1.00 (Ref.)
pNx	1.19 (1.08–1.30)***	1.05 (0.86–1.29)	1.20 (0.98–1.48)	1.15 (1.01–1.31)*	1.59 (1.20–2.11)**
pN1–3	2.20 (1.93–2.50)***	2.13 (1.33–3.41)**	2.33 (1.60–3.40)***	2.00 (1.68–2.39)***	2.26 (1.65–3.10)***

*P < 0.05, **P < 0.01, ***P < 0.001. ^a^ Adjusted to age, gender, race, tumor location, laterality, tumor size, tumor stage, tumor grade, chemotherapy and year of surgery. ^b^ Adjusted to age, gender, race, tumor location, laterality, tumor size, tumor grade, chemotherapy and year of surgery. *HR* hazard ratio, *95% CI* 95% confidence interval, *LND* lymph node dissection

**Table 4:** Multivariable Cox regression analyses predicting cancer-specific survival

	All stage	T1	T2	T3	T4
	Multivariable ^a^ HR (95% CI)	Multivariable ^b^ HR (95% CI)	Multivariable ^b^ HR (95% CI)	Multivariable ^b^ HR (95% CI)	Multivariable ^b^ HR (95% CI)
LND status					
No LND	1.00 (Ref.)	1.00 (Ref.)	1.00 (Ref.)	1.00 (Ref.)	1.00 (Ref.)
LND	0.81 (0.73–0.95)**	0.84 (0.56–1.25)	0.87 (0.61–1.24)	0.83 (0.73–0.98)*	0.64 (0.47–0.88)**
LND extent					
No LND	1.00 (Ref.)	1.00 (Ref.)	1.00 (Ref.)	1.00 (Ref.)	1.00 (Ref.)
Limited LND	0.86 (0.75–0.96)*	1.02 (0.65–1.59)	1.02 (0.69–1.51)	0.92 (0.78–1.16)	0.61 (0.43–0.87)**
Extended LND	0.73 (0.61–0.88)**	0.56 (0.29–1.08)	0.65 (0.38–1.12)	0.74 (0.58–0.95)*	0.70 (0.47–0.96)*
Lymph node stage					
pN0	1.00 (Ref.)	1.00 (Ref.)	1.00 (Ref.)	1.00 (Ref.)	1.00 (Ref.)
pNx	1.32 (1.13–1.53)***	1.29 (0.85–1.98)	1.19 (0.83–1.72)	1.22 (1.01–1.49)*	1.97 (1.31–2.97)**
pN1–3	2.49 (2.06–3.01)***	3.40 (1.61–7.18)**	3.35 (1.87–5.99)***	2.32 (1.81–2.98)***	2.55 (1.64–3.98)***

*P < 0.05, **P < 0.01, ***P < 0.001. ^a^ Adjusted to age, gender, race, tumor location, laterality, tumor size, tumor stage, tumor grade, chemotherapy and year of surgery. ^b^ Adjusted to age, gender, race, tumor location, laterality, tumor size, tumor grade, chemotherapy and year of surgery. *HR* hazard ratio, *95% CI* 95% confidence interval, *LND* lymph node dissection

## Discussion

Our study aimed to determine the impact of LND on survival in UTUC patients following NU. In this study, LND was performed in only 26.9% of the UTUC patients, which seemed to be infrequent compared to the LND rate 75.0% at radical cystectomy (RC) for non-metastatic bladder transitional cell carcinoma [8]. While, LND was increasingly considered at NU year by year from 2004 (23.5%) to 2015 (34.1%), which indicated that urologists were increasingly aware of the crucial role of LND. And this trend was consistent with a previous UTUC study performed by Chappidi et al. [9]. Moreover, younger patients, non-white race, left laterality, bigger tumor (> 2 cm), higher grade and advanced tumor stage were important factors contributing to the decision-making of LND. Kaplan-Meier plots illustrated that extended LND with 4 or more regional lymph nodes removed might benefit OS or CSS for pT1-pT3 and limited LND with 1 to 3 regional lymph nodes removed may benefit OS or CSS for pT4 disease, although not to a significant extent (Fig. 2, Additional file 1: Figure S2 and Figure S3). However, in multivariable Cox regression analyses, LND was beneficial for UTUC patients especially for pT3 and pT4 disease. Extended LND brought evident benefits to pT3 and pT4 patients, while limited LND was only beneficial for pT4 patients and more effective than extended LND. These findings suggested LND could be considered at NU for pT3/4, for pT1/2 remains to be seen, both of which would be verified by further prospective studies.

At present, the therapeutic role of LND at NU for UTUC patients remains controversial [10–13]. Most studies worked on the effect of LND on survival in all stage patients. However, LND was seldom considered unless preoperative examinations indicated a high probability of tumor invasiveness or LNM. Therefore, the patients undergoing LND were prone to worse pathologic tumor stage compared to those without LND, which resulted in the bias for survival analysis. As a result, we performed this stage-specific study to eliminate the bias and investigate whether LND could bring benefits to survival. Also, we included the extent of LND in the analysis since previous studies had stressed its clinical role [8, 9, 14].

Firstly, the LND rate was evidently increasing from 2004 to 2015 (Additional file 1: Figure S1), which indicated that urologists were likely to perform LND at NU for UTUC patients. A recent study performed by Chappidi et al. [9] described a similar increasing trend from 20% in 2004 to 33% in 2012 for UTUC patients. Similar trends were also found in other cancers [8, 15], which suggested LND was becoming more and more important in cancer therapy. Secondly, LND were likely to be performed in younger, non-white race, left laterality, bigger tumor (> 2 cm), higher grade and advanced tumor stage patients according to this study, which may due to the good surgical tolerance of young patients and higher probability of aggressive tumor in these patients. Then, we found that LND might improve OS and CSS across all tumor stages especially for pT3 to pT4 disease from Kaplan-Meier plots and COX regression analysis. To further investigate the effect of LND extent on the prognosis, we divided the LND population into two groups: limited LND and extended LND. The plots described patients who underwent LND with 4 or more regional lymph node removed survived longer than those in pT1 to pT3 stages. While, patients receiving LND with 1 to 3 regional lymph nodes removed survived the longest in pT4 stage. In COX analysis, extended LND improved survival significantly in pT3 to pT4 disease, while limited LND only improved the survival of pT4 patients evidently and it seemed more beneficial than extended LND. This may due to the higher incidence of severe complications resulting from extended LND in pT4. However, this may also due to selection bias. Anyway, it needs to be verified by further prospective studies. In the past few years, there were studies working on the effect of LND for UTUC patients. Sebastiano Nazzani et al. reported that LND could provide extensive prognostic information [16]. According to Fan Dong et al. [13], LND could improve survival for clinically node-negative UTUC patients, and its protective role seemed to be more evident in muscle-invasive diseases. While, the study conducted by Inokuchi Junichi et al. indicated that there was no therapeutic benefit of LND for UTUC patients treated with NU [12]. Inokuxhi Junichi et al. did not stratify the patients according to tumor stage, thus patients with LND were more likely to suffer clinically node-positive or muscle-invasive diseases, which might result in the bias. In our study, LNM rates were 12.2, 20.3, 39.4 and 65.3% for pT1, pT2, pT3 and pT4 patients and 19.8, 86.5, 93.4 and 85.0% for N0, N1, N2 and N3 patients respectively, which indicated that the stages of patients with LND were more advanced. Zareba Piotr et al. showed that a higher LN yield was associated with lower all-cause mortality [17]. Winer AG et al. consistently reported that extended LND might have a beneficial effect on oncologic outcomes in high-risk UTUC patients without increasing surgical time or risk of complications [18]. Consequently, LND especially extended LND at NU should particularly be contemplated in locally advanced UTUC patients, as the protective effect of LND on OS or CSS is more evident in these patients compared to organ-confined diseases. Furthermore, we observed that LND might have a minimal positive impact on OS or CSS in pT1/2 patients. This may result from the difficulty to standardize indication or template for LND [4, 19]. In consequence, it simply cannot be argued that LND is of no benefit for organ-confined UTUC patients.

Additionally, our results validated the detrimental impact of LNM across all tumor stages. Patients who did not receive LND (pNx) fared worse than those with absence of LNM confirmed by LND (pN0). Patients with LNM (pN1–3) validated by LND fared the worst. And these results were consistent across all stages (Tables 3 and 4). Similar studies have also reported that patients with pNx might have a worse prognosis compared to those with pN0 [20, 21]. Therefore, LND should be recommended as long as it can be safely performed.

Several limitations of the study should be acknowledged. This study was performed in an observational manner. And the groups might differ in recorded or unrecorded variables, which may influence survival, since patients were not randomized to receive LND. Moreover, the anatomical extent of LND could not be defined since the extent of LND lacks standardization, and the number of regional lymph nodes removed was used instead as a proxy. Different treatment groups, surgeon experience, approach to radical nephroureterectomy and academic vs non-academic facility might also result in selection bias.

## Conclusions

LND was more frequently performed in locally advanced UTUC patients. The beneficial effect of LND especially LND with 4 or more regional lymph nodes removed on survival was evident in pT3/4 disease, while pT1 and pT2 fell out in terms of benefit of LND. LND with 1 to 3 regional lymph nodes removed seemed to be more beneficial than LND with 4 or more regional lymph nodes removed in pT4 disease. LND can be considered for pT3 and pT4, for pT1/2 remains to be seen, both of which will be verified by further prospective studies.

## Supplementary information

**Additional file 1: Table S1.** Year of diagnosis for UTUC patients stratified by lymph node dissection. **Table S2.** Descriptive characteristics of 1961 LND patients according to T stage. **Table S3.** Extent of LND in the same stage disease. **Table S4.** AJCC N stage distribution according to LND status. **Figure S1.** Trend of LND rate from 2004 to 2015. **Figure S2.** Trend of 5-year OS rate across all tumor stages according to LND status. **Figure S3.** Trend of 5-year CSS rate across all tumor stages according to LND status.

## Data Availability

The datasets used and/or analyzed during this current study are available from the corresponding author on reasonable request.

## Abbreviations

CSS: Cancer-specific survival
LND: Lymph node dissection
LNM: Lymph node metastasis
NU: Nephroureterectomy
OS: Overall survival
SEER: Surveillance, epidemiology and end results database
UTUC: Upper urinary tract urothelial carcinoma
