# Supplementary material for: Effect of lymph node dissection on stage-specific survival in patients with upper urinary tract urothelial carcinoma treated with nephroureterectomy
Source: BMC Cancer. 2019 Dec 12;19:1207. doi: 10.1186/s12885-019-6364-z (PMC6907152; doi:10.1186/s12885-019-6364-z)
Supplement: Supplementary file 1 — Additional file 1: Table S1. Year of diagnosis for UTUC patients stratified by lymph node dissection. Table S2. Descriptive characteristics of 1961 LND patients according to T stage. Table S3. Extent of LND in the same stage disease. Table S4. AJCC N stage distribution according to LND status. Figure S1. Trend of LND rate from 2004 to 2015. Figure S2. Trend of 5-year OS rate across all tumor stages according to LND status. Figure S3. Trend of 5-year CSS rate across all tumor stages according to LND status. [file 12885_2019_6364_MOESM1_ESM.docx]

**Effect of lymph node dissection on stage-specific survival in patients with upper urinary tract urothelial carcinoma treated with nephroureterectomy.**

Table S1 Year of diagnosis for UTUC patients stratified by lymph node dissection.

|  | Total* | No LND* | LND* | *P value*‡ |
| --- | --- | --- | --- | --- |
| Characteristic | No. (%) | No. (%) | No. (%) |  |
| Year of diagnosis |  |  |  | <0.001 |
| 2004 | 587 (8.1) | 449 (76.5) | 138 (23.5) |  |
| 2005 | 585 (8.0) | 445 (76.1) | 140 (23.9) |  |
| 2006 | 653 (9.0) | 513 (78.6) | 140 (21.4) |  |
| 2007 | 599 (8.2) | 458 (76.5) | 141 (23.5) |  |
| 2008 | 616 (8.5) | 458 (74.4) | 158 (25.6) |  |
| 2009 | 648 (8.9) | 468 (72.2) | 180 (27.8) |  |
| 2010 | 621 (8.5) | 455 (73.3) | 166 (26.7) |  |
| 2011 | 566 (7.8) | 422 (74.6) | 144 (25.4) |  |
| 2012 | 558 (7.7) | 388 (69.5) | 170 (30.5) |  |
| 2013 | 602 (8.3) | 422 (70.1) | 180 (29.9) |  |
| 2014 | 581 (8.0) | 403 (69.4) | 178 (30.6) |  |
| 2015 | 662 (9.1) | 436 (65.9) | 226 (34.1) |  |

*With percentages in parentheses; ‡Fisher’s exact test or χ2 test. LND, Lymph Node Dissection.

Table S2 Descriptive characteristics of 1961 LND patients according to T stage.

|  | Total (%) | pN0 (%) | pN1-3 (LNM) (%) | P value |
| --- | --- | --- | --- | --- |
|  | 1961 (100) | 1296 (66.1) | 665 (33.9) |  |
| LND |  |  |  | 0.008 |
| Limited LND | 1108 (56.5) | 760 (68.6) | 348 (31.4) |  |
| Extended LND | 853 (43.5) | 536 (62.8) | 317 (37.2) |  |
| T stage |  |  |  | < 0.001 |
| pT1 | 425 (21.7) | 373 (87.8) | 52 (12.2) |  |
| pT2 | 315 (16.1) | 251 (79.7) | 64 (20.3) |  |
| pT3 | 959 (48.9) | 581 (60.6) | 378 (39.4) |  |
| pT4 | 262 (13.4) | 91 (34.7) | 171 (65.3) |  |
| T stage |  |  |  |  |
| pT1 |  |  |  | 0.048 |
| Limited LND | 234 (55.1) | 212 (90.6) | 22 (9.4) |  |
| Extended LND | 191 (44.9) | 161 (84.3) | 30 (15.7) |  |
| pT2 |  |  |  | 0.946 |
| Limited LND | 176 (55.9) | 140 (79.5) | 36 (20.5) |  |
| Extended LND | 139 (44.1) | 111 (79.9) | 28 (20.1) |  |
| pT3 |  |  |  | 0.022 |
| Limited LND | 546 (56.9) | 348 (63.7) | 198 (36.3) |  |
| Extended LND | 413 (43.1) | 233 (56.4) | 180 (43.6) |  |
| pT4 |  |  |  | 0.058 |
| Limited LND | 152 (58.0) | 60 (39.5) | 92 (35.1) |  |
| Extended LND | 110 (42.0) | 31 (28.2) | 79 (71.8) |  |

LND, Lymph Node Dissection; LNM, Lymph Node Metastases.

Table S3 Extent of LND in the same stage disease.

|  | Limited LND | Extended LND | P value |
| --- | --- | --- | --- |
| T stage |  |  | 0.867 |
| pT1 | 234 (55.1) | 191 (44.9) |  |
| pT2 | 176 (55.9) | 139 (44.1) |  |
| pT3 | 546 (56.9) | 413 (43.1) |  |
| pT4 | 152 (58.0) | 110 (42.0) |  |
| AJCC N stage |  |  | < 0.001 |
| N0 | 760 (58.6) | 536 (41.4) |  |
| N1 | 235 (64.2) | 131 (35.8) |  |
| N2 | 108 (38.3) | 174 (61.7) |  |
| N3 | 5 (29.4) | 12 (70.6) |  |

LND, Lymph Node Dissection. AJCC N stage refers to AJCC 6^th^ ed.

Table S4 AJCC N stage distribution according to LND status.

|  | Total (%) | No LND (%) | LND (%) | P value |
| --- | --- | --- | --- | --- |
| AJCC N stage |  | 5317 | 1961 | < 0.001 |
| N0 | 6533 (89.8) | 5237 (80.2) | 1296 (19.8) |  |
| N1 | 423 (5.8) | 57 (13.5) | 366 (86.5) |  |
| N2 | 302 (4.1) | 20 (6.6) | 282 (93.4) |  |
| N3 | 20 (0.3) | 3 (15.0) | 17 (85.0) |  |

LND, Lymph Node Dissection.

Figure S1 Trend of LND rate from 2004 to 2015.

LND, Lymph Node Dissection.

Figure S2 Trend of 5-year OS rate across all tumor stages according to LND status.

LND, Lymph Node Dissection.

Figure S3 Trend of 5-year CSS rate across all tumor stages according to LND status.

LND, Lymph Node Dissection.
